# Class-dependent and cross-modal memory network considering sentimental features for video-based captioning

**DOI:** 10.3389/fpsyg.2023.1124369

**Published:** 2023-02-15

**Authors:** Haitao Xiong, Yuchen Zhou, Jiaming Liu, Yuanyuan Cai

**Affiliations:** ^1^School of International Economics and Management, Beijing Technology and Business University, Beijing, China; ^2^National Engineering Research Centre for Agri-Product Quality Traceability, Beijing Technology and Business University, Beijing, China; ^3^School of E-Business and Logistics, Beijing Technology and Business University, Beijing, China

**Keywords:** cross-modal mapping, cross-modal memory network, commonsense caption, cross-modal matrices, sentimental features, class-dependent memory

## Abstract

The video-based commonsense captioning task aims to add multiple commonsense descriptions to video captions to understand video content better. This paper aims to consider the importance of cross-modal mapping. We propose a combined framework called Class-dependent and Cross-modal Memory Network considering SENtimental features (CCMN-SEN) for Video-based Captioning to enhance commonsense caption generation. Firstly, we develop class-dependent memory for recording the alignment between video features and text. It only allows cross-modal interactions and generation on cross-modal matrices that share the same labels. Then, to understand the sentiments conveyed in the videos and generate accurate captions, we add sentiment features to facilitate commonsense caption generation. Experiment results demonstrate that our proposed CCMN-SEN significantly outperforms the state-of-the-art methods. These results have practical significance for understanding video content better.

## 1. Introduction

**Figure 1:**
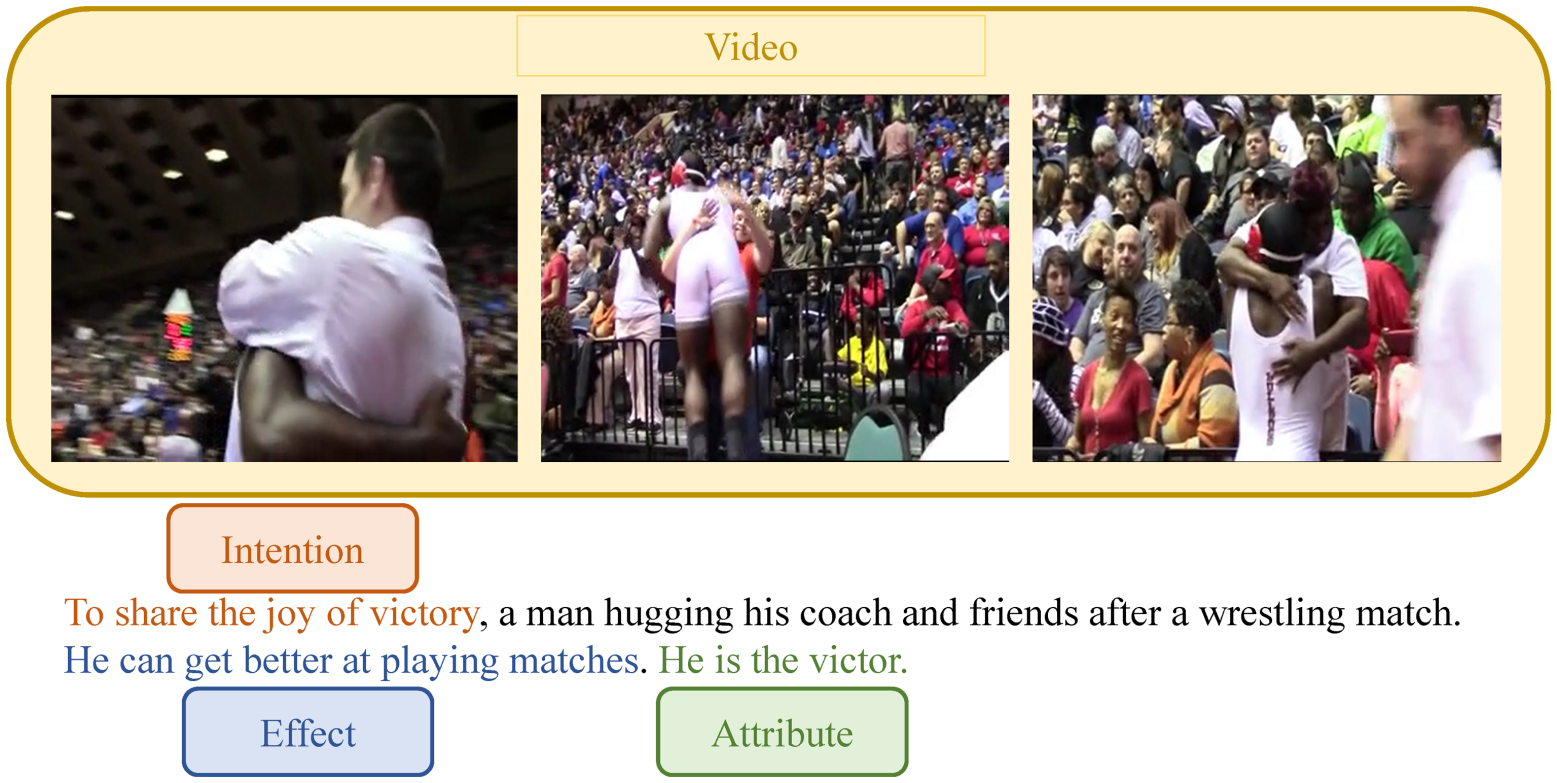
The example of the video-based commonsense caption generation.

Progress has been made in describing human activity in videos thanks to significant advances in deep learning. However, most research (Ramanishka et al., 2017; Tan et al., 2020; Wang et al., 2019; Zhang H. et al., 2020) efforts to date have focused on identifying objects and actions and thus composing sentences that describe events. Its task is to provide one or more text descriptions that correspond to the content of the video. The generated captions can be used for video retrieval in the future and directly assist visually impaired people in understanding reality. Recently, research on video-based commonsense captioning (Fang et al., 2020; Yu et al., 2021) has gained traction, which can reason about the underlying aspects of the video rather than simply describing the events in the video. Given an input video, the video-based commonsense captioning task aims to simultaneously generate captions and three types of commonsense descriptions (intention, effect, attribute). An example is shown in Figure 1. When humans watch the video, they can not only describe the event of “a man hugging his coach and friends after a wrestling match,” but also understand the intention of “to share the joy of victory,” the effect of “getting better at playing matches” and the attribute “victor.”

Video-based commonsense captioning is a cutting-edge research topic, and Video2Commonsense (Fang et al., 2020) executes separate networks to learn different types of commonsense separately. The current best-performing HybridNet (Yu et al., 2021) learns from various sources of information through multi-modal fusion based on multiple commonsense semantic interactions and records historical prediction sequences through memory modules. Although these methods have achieved remarkable results, we believe they need to be improved in three aspects. Firstly, they have limited ability to make full use of the information in videos and captions. The limitation comes from focusing on semantic relationships between texts while ignoring video-text correspondences and the lack of suitable model design to learn cross-modal correspondences. Existing methods focus on learning the semantic relationships between texts when generating captions while neglecting the importance of cross-modal interactions, which are critical for dealing with complex video and text semantic relationships. Each word in a human description of a specific event is associated with the previous word and video information. Second, they did not focus on the effect of video type on the results. Humans’ commonsense descriptions of videos of the same type will be more similar and consistent. There may be noise in the interaction between different types of videos and text. We should consider commonsense reasoning in a broader sense, considering the interaction between other modalities and the effect of video type on the results.

Furthermore, compared with image captions, video captions usually show more complex semantic patterns that contain more sentimental features. Previous studies only focused on the characters’ behavior in the video and ignored their sentimental attributes. Sentiment is a critical component of user-generated videos. Sentimental factors are present in human activities in videos. The sentimental content of videos can be used to help create commonsense descriptions. When a video’s sentiment is positive, we can easily infer that the generated description should also be positive. However, the current study does not consider sentimental features, instead relying solely on motion features. The recent research may result in the predicted words being biased or taking longer to correct.

**Figure 2:**
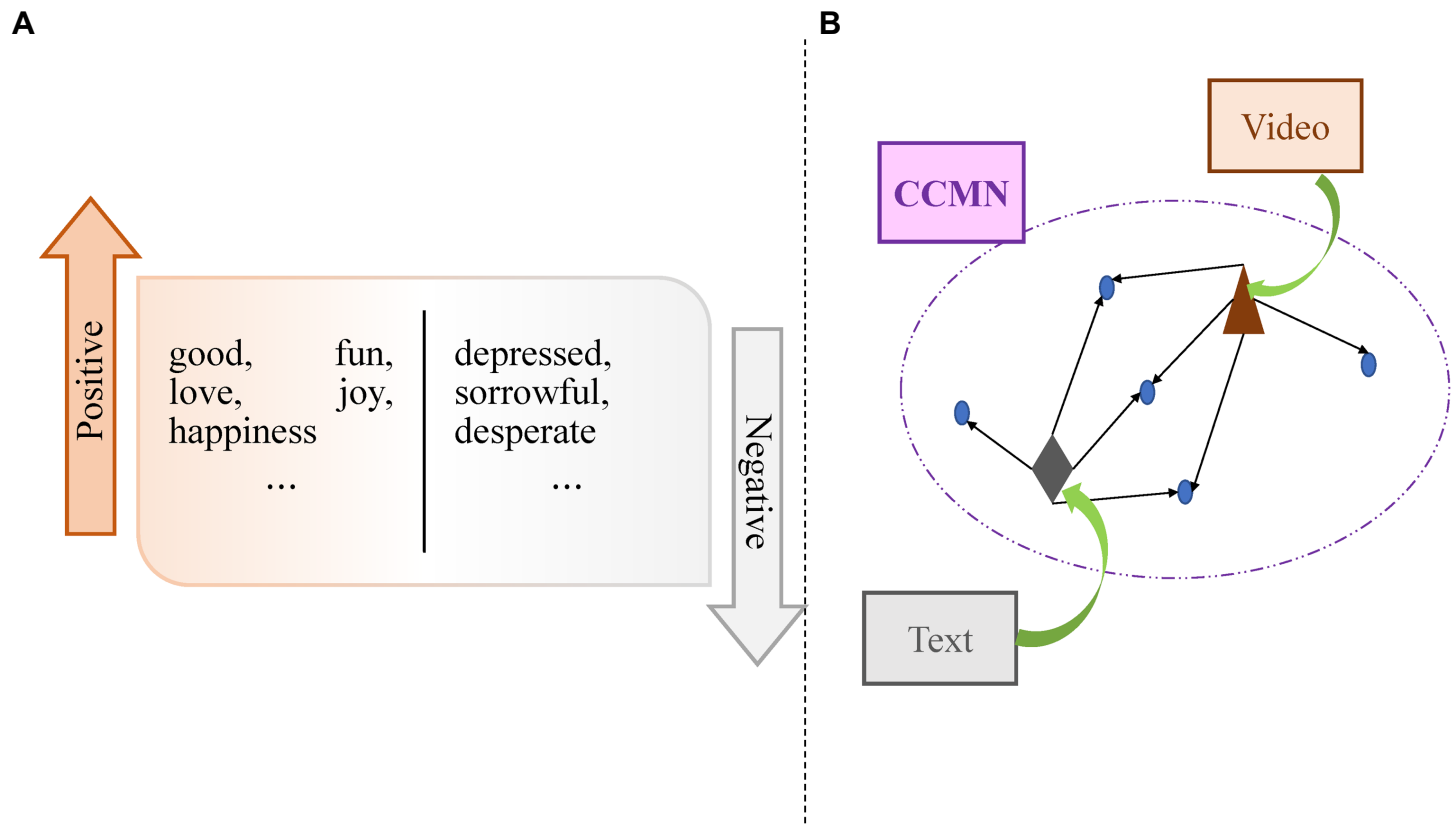
**(A)** We implement sentiment control which learns to generate texts corresponding to sentiments given bipolar sentiment types. **(B)** Cross-modal interaction makes the aligned video and text features point to the same content.

To address the abovementioned issues, we implement sentiment control in Figure 2A, which learns to generate texts corresponding to bipolar sentiment types. Then, we implement cross-modal interaction in Figure 2B, making the aligned video and text features point to the same content. Specifically, the class-dependent cross-modal memory network (CCMN) is our memory. The triangle and rhombus represent video and text features within the same representation space. Each node is a storage vector. The arrow indicates that information is stored in this node, and the shared information of video and text features will point to the same node. The whole process can be summarized as follows: the shared information of video and text features is recorded in memory so that the whole learning process can be explicitly mapped between video and text. In this paper, we propose a novel Class-dependent and Cross-modal Memory Network considering SENtimental features (CCMN-SEN) framework by cleverly integrating them into our model. Experimental results on the benchmark dataset V2C confirm the effectiveness and efficiency of our proposed model, achieving state-of-the-art performance. Ablation experiments and example visualizations are also performed to analyze the impact of different components of our model and to show that our model can generate commonsense captions.

For sentiment control, we use bipolar sentiment and the sentimental content in the video to facilitate text generation corresponding to sentiment. As a control variable (a given condition), sentiment encourages the model to generate text that matches the control variable. Our model uses sentiment control to go from positive to negative. For example, if the given control variable is positive in sentiment, the model will make generating text that reflects positive sentiments easier. The sentiment feature we introduced represents the SENtimental Dimension (SD). Furthermore, we use multi-modal fusion to combine 1D audio features, 2D appearance features, 3D motion features, and SD sentiment features, allowing our model to learn to generate commonsense captions from different sources of information.

To consider the interaction between different modalities and the importance of video type, we propose a class-dependent cross-modal memory network to record the alignment of video and text to facilitate interaction between modalities. Specifically, we use prior information to initialize a shared memory network matrix and use it to perform memory queries and memory responses on video and text features. For memory queries, we measure the similarity of cross-modal features and memory vectors under the same label and select the vectors with the highest similarity to interact with cross-modal features, and calculate their weights. The memory responses are generated by weighting the memory vector of the query and then feeding the responses corresponding to the input video and text features to the encoder and decoder to generate commonsense captions from learned interactions of cross-modal information.

Our contributions are summarized below:

- We propose a combined framework called Class-dependent and Cross-modal Memory Network considering SENtimental features (CCMN-SEN) to generate relevant commonsense captions.
- A class-dependent cross-modal memory network is proposed to record the alignment of video and text to facilitate interaction between modalities.
- Sentimental Dimension (SD) is introduced, making generating text corresponding to video sentiment easier.
- Extensive experiments and analyses demonstrate the effectiveness and superiority of our proposed model.

## 2. Related work

### 2.1. Commonsense knowledge

In recent years, commonsense knowledge has increasingly become a research hotspot in the field of NLP and even multi-modal (Jin et al., 2016; Zhang and Peng, 2019) and interdisciplinary. Pre-trained language models represented by BERT (Devlin et al., 2018) have achieved outstanding performance in tasks such as entity recognition, machine translation, and sentiment analysis. Integrating commonsense knowledge into machine learning has become a promising solution. More and more researchers are focusing on visual understanding by targeting visual commonsense reasoning (Ostermann et al., 2018; Rashkin et al., 2018; Talmor et al., 2018; Tandon et al., 2018). Commonsense-based reasoning tasks have multiple datasets (Luo et al., 2016; Speer et al., 2017; Bosselut et al., 2019; Zhang Y. et al., 2020) for reasoning about various types of commonsense. Zellers et al. (2019) proposed a visual commonsense reasoning task that not only provides a question answer but also predicts the correct rationale behind the answer based on the question and images. Recently, commonsense-based text generation has been explored through the ATOMIC dataset (Sap et al., 2019), a corpus of 877 k textual descriptions of inference knowledge organized into relations. For video-based commonsense captioning, Fang et al. (2020) innovatively use the ATOMIC dataset to generate commonsense descriptions from visual input. Compared to these studies, we propose a combination model to add multiple commonsense descriptions to video captions. It leverages cross-modal information flow and sentimental features to understand video content better, resulting in more accurate captions.

### 2.2. Video captioning

Captioning is essential for understanding visual effects. The task of video captioning (Guo et al., 2019; Shi et al., 2020; Xu et al., 2021; Zhang et al., 2021) is to imitate human learning to connect vision and language. Usually, video captions simply describe observable objects and events in one sentence. To develop video captioning, some researchers tend to use open-domain video captioning datasets (Das et al., 2013; Xu et al., 2016). Recently, Fang et al. (2020) attempted to link video captions with commonsense, exploring commonsense descriptions in videos and proposing a dataset named V2C (Fang et al., 2020). Furthermore, current deep learning-based video captioning performs sequence-to-sequence learning in the codec paradigm. Zhou et al. (2018) use the CNN features of the frame-by-frame image to the transformer and then input it to the decoder through TCN to generate captions. Jin et al. (2016) use all the available data to perform multi-modal fusion through a fusion network and then input it to the decoder side to get captions. Zhang and Peng (2019) extract the video’s key objects, build the bidirectional sequence diagram optimization features, and finally fuse the global features to generate captions. Zhong et al. (2020) generate captions by sampling the scene graph obtained from the image by multiple subgraphs, and then the subgraphs are used to generate captions. Fang et al. (2020) use an encoder-decoder approach to model specific generic captioning individually without using commonsense correlation, which lacks commonsense interactivity. Yu et al. (2021) work by generating commonsense descriptions in videos from semantic-level and word-level reasoning. It adds commonsense relevance to the model but focuses on the interaction between text and text and ignores the interaction between video and text. Different from these works, we use a class-dependent cross-modal network and sentimental features to facilitate the generation of commonsense video captions. Specifically, we first improved the memory network module to record the cross-modal alignment relationship between video and text rather than simply recording the relationship between text. Secondly, our query and response processes are class related. Cross-modal learning is only performed on the shared matrix with the same label, not in all matrices. Finally, we introduce the sentimental features of video ignored by previous studies and fuse them with appearance, motion, and audio features to help the model better understand the video content. Compared with other studies, we consider the interaction between different modalities, the effect of video type on the results, and the influence of sentimental features. So, our model can generate more accurate descriptions of commonsense.

## 3. Class-dependent and cross-modal memory network considering sentimental features

**Figure 3:**
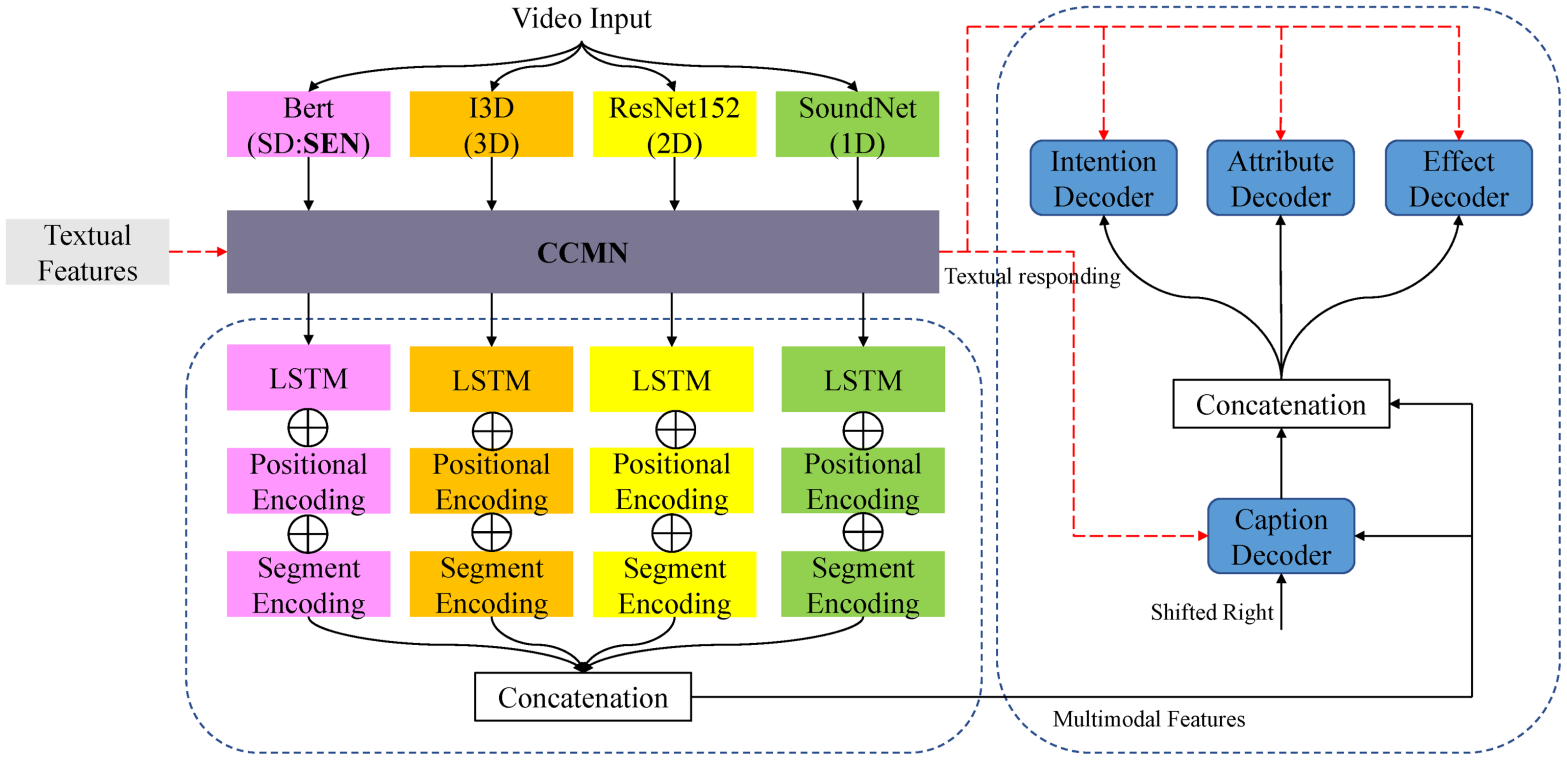
The overall architecture of our proposed CCMN-SEN includes a video encoder, a cross-modal memory network, a caption decoder, and three common sense decoders. Video and text features can be recorded in the memory network to form a mapping between video and text.

This section describes the architecture and design of our proposed CCMN-SEN. It records the alignment between video features and text, allowing cross-modal interactions and generation to take place on cross-modal matrices that share the same labels, facilitates cross-modal interactions, and leverages sentiment to facilitate commonsense captioning generation. As illustrated in Figure 3, our CCMN-SEN is an encoder-decoder architecture that includes a video encoder, a class-dependent cross-modal memory network, a caption decoder, and three commonsense decoders. Given a video input, there are four pre-trained models to extract multiple features, including audio features (1D), appearance features (2D), motion features (3D), and SENtiment features (SD). Then the features are sent to a class-dependent cross-modal memory network module (CCMN) to measure the similarity between their feature representation. The cross-modal model memory vector under the same label as the video selects the top vector with the highest similarity to interact with the feature representation, obtain memory responses, and apply a linear layer to integrate video features and memory responses. Secondly, the multi-modal fusion method combines the extracted features into multi-modal features. Input the ground truth captions into the caption decoder to obtain the caption encoding. The commonsense decoder then uses multi-modal features and caption encoding as input to generate the commonsense description. Finally, we send the text features to the class-dependent cross-modal memory network to obtain memory responses. It is worth noting that our sentiment features make generating sentiment words that match easier.

Consider a video *V* consisting of what is described in sentence *S*. Our framework can be used for generating commonsense descriptions C in the setting. In the setting (Completion task), we use ground-truth captions to guide the generation of commonsense captions. This task can be seen as a complement to captions. The setting can be formulated as:


(1) $$C_{com}=f\left(V,S\right)$$

where *V* is the video and *S* is the ground truth caption.

The following subsections detail and discuss our contributions, which primarily focus on the design of class-dependent cross-modal memory networks.

### 3.1. Encoder

Given a video, we use pre-trained models, including ResNet152 (He et al., 2016), SoundNet (Aytar et al., 2016), I3D (Carreira and Zisserman, 2017), and Bert (Devlin et al., 2018), to extract appearance features, audio features, motion features, and sentiment features, respectively. We use feature-level fusion to fuse the information extracted from each modality, which avoids scaling and normalizing features because fusion involves concatenation and no overlapping, merging, or combining. As shown in Figure 3, we feed the features to the cross-modal memory network to obtain memory responses, use LSTM (Hochreiter and Schmidhuber, 1997) to encode different features separately, and utilize the last hidden state of the LSTM as the final representation. Finally, we concatenate multi-modal features by adding customized positional and segment encodings to the final representation. Take the appearance features, the concatenating process can then be formulated as:


(2) $$E^{2D}=LSTM\left(V^{2D}\right)+SE^{2D}+PE^{2D}$$

where $E^{2D}$ is the encoded appearance feature, and $V^{2D}$ is the appearance feature. The $SE^{2D}$ is 2D segment encoding, and $PE^{2D}$ means 2D positional encoding. Similarly, we can obtain the encoded audio feature, encoded motion feature, and encoded sentiment feature. Then we can concatenate them to get multi-modal features using a multi-modal fusion method.

### 3.2. Decoder

The video encoding is fed into two transformer language decoder networks (a robust architecture that achieves state-of-the-art on many tasks). Our commonsense decoder network uses multi-commonsense learning similar to Yu et al. (2021) to improve each commonsense semantics’ advanced reasoning ability. Our model predicts the current event directly from the video and then generates the commonsense captions to go with it. The caption decoder uses video encoding and ground truth caption as input to generate caption encodings. In contrast, the commonsense decoder uses the video and caption encodings to generate commonsense descriptions. The memorized responses of caption and commonsense features are functionalized as inputs to the decoder networks to improve the generation process.

### 3.3. Sentimental dimension

Video provides humans with an emerging channel to express sentiments, which play a crucial role in human life. These sentiments can be defined as complex psychological states such as anger, disgust, amusement, awe, etc. Sentiments can be positive or negative, and they belong to different sentiment categories (positive or negative). In this paper, we introduce Sentiment Dimension (SD) in the preprocessing stage, aiming to help create commonsense descriptions. The SD represents the SEN in our proposed model. Specifically, we use the pre-trained model Bert to get each video’s sentiment category (positive or negative) and then convert the sentiment category into a 768-dimensional sentiment representation. The resulting representation is used as the source input for all subsequent modules.

### 3.4. Class-dependent cross-modal memory networks

There could be correlations between different modalities of video and text for the video-to-commonsense captioning task. These associations can be an excellent reference to aid in the generation process. It is also possible to record the alignment of cross-modal representations such as video and text. At the same time, there may be noise in the interaction between different types of video and text, and it can be more accurate only to allow interaction between the same type of video and text. To take advantage of this relationship, we use CCMN to improve the mapping relationship, make cross-modal video and text interaction more effortless, and make commonsense caption generation easier.

**Figure 4:**
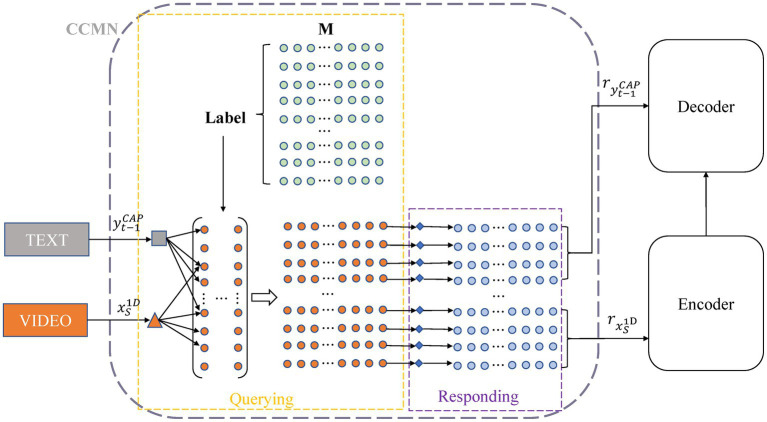
Our cross-modal memory network records video features and text features, and the yellow and purple dashed boxes show the process of memory query and response, respectively.

As shown in Figure 4, given a source sequence $\left\{x_{1}^{1D},x_{2}^{1D},\cdots ,x_{S}^{1D}\right\}$ from the audio feature, we feed it to this module to obtain the memory responses of the audio features $\left\{r_{x_{1}^{1D}},r_{x_{2}^{1D}},\cdots ,r_{x_{S}^{1D}}\right\}$. Similarly, we can obtain the memory responses from the appearance, motion, and sentiment features. Given a generated caption sequence $\left\{y_{1}^{CAP},y_{2}^{CAP},\cdots ,y_{t-1}^{CAP}\right\}$, it is also fed to the cross-modal memory networks to output the memory responses of the textual features $\left\{r_{y_{1}^{CAP}},r_{y_{2}^{CAP}},\cdots ,r_{y_{t-1}^{CAP}}\right\}$. We also can obtain the textual responses from the commonsense description. The cross-modal memory network uses the matrix to store the information from the encoding and decoding processes. Each row of the matrix records certain cross-modal information connecting videos and texts. We denote the matrix as $M=\left\{m_{1},m_{2},\cdots ,m_{i},\cdots ,m_{N}\right\}$, where N represents the number of memory vectors, and $m_{i}\in {\mathbb{R}}^{d}$ indicates the memory vector at row i with d referring to its dimension.

Instead of randomly initializing matrices, as in previous studies, which may hinder subsequent matrix learning, we utilize prior information. Specifically, for the same type of video, we utilize pre-trained models to extract video and text features separately and combine them, then employ K-Means to cluster each feature set into $N_{p}\;$clusters and use the mean of the features in each cluster as the initial value in the matrix. CCMN operates with two main steps during the report generation process, namely querying and responding, which are described in detail below.

#### 3.4.1. Memory querying

Our cross-modal matrix query first measures the similarity between its feature representation and the cross-modal model memory vector under the same label as the video and selects the top vector with the highest similarity to interact with the feature representation. We apply multi-thread querying to perform this operation, wherein each thread of the querying process follows the same procedure. We linearly transform each memory vector in M and the input features to ensure the input video and text features are in the same representation space. Taking audio features and caption text features as examples, the linear transform process is formalized as:


(3) $$k_{i}=m_{i}\cdot W_{k}$$

(4) $$q_{s}^{1D}=x_{s}^{1D}\cdot W_{q}$$

(5) $$q_{t}^{CAP}=y_{t}^{CAP}\cdot W_{q}$$

where $W_{k}$ and $W_{q}$ are trainable weights for the conversion. Then according to the distance of video features and text features, the most relevant memory vectors are extracted respectively:


(6) $$A_{s_{i}}^{1D}=\frac{q_{s}^{1D}\cdot k_{i}^{⊺}}{\sqrt{d}}$$

(7) $$A_{t_{i}}^{CAP}=\frac{q_{t}^{CAP}\cdot k_{i}^{⊺}}{\sqrt{d}}$$

where the number of extracted memory vectors can be controlled by a hyper-parameter K to regularize how much memory is used. We only select the most similar vector to respond to the query vector. We denote the queried memory vectors as $\left\{k_{s_{1}},k_{s_{2}},\cdots ,k_{s_{j}},\cdots ,k_{s_{K}}\right\}$ and $\left\{k_{t_{1}},k_{t_{2}},\cdots ,k_{t_{j}},\cdots ,k_{t_{K}}\right\}$. Afterward, the importance weight of each memory vector concerning audio and caption text features is obtained by normalization over all distances by:


(8) $$W_{s_{i}}^{1D}=\frac{\exp\left(A_{s_{i}}^{1D}\right)}{\sum_{j=1}^{K}\exp\left(A_{s_{j}}^{1D}\right)}$$

(9) $$W_{t_{i}}^{CAP}=\frac{\exp\left(A_{t_{i}}^{CAP}\right)}{\sum_{j=1}^{K}\exp\left(A_{t_{j}}^{CAP}\right)}$$

#### 3.4.2. Memory responding

The responding process is also conducted in a multi-thread manner corresponding to the query process. For each thread, we first perform a linear transformation on the queried memory vector *via*:


(10) $$v_{i}=m_{i}\cdot W_{v}$$

where $W_{v}$ is the trainable weight for $m_{i}$. So that all memory vectors $\left\{\nu_{s_{1}},\nu_{s_{2}},\cdots ,\nu_{s_{j}},\cdots ,\nu_{s_{K}}\right\}$ are transferred into $\left\{\nu_{t_{1}},\nu_{t_{2}},\cdots ,\nu_{t_{j}},\cdots ,\nu_{t_{K}}\right\}$. Then, we obtain the memory responses for audio and caption text features by weighting over the transferred memory vectors:


(11) $$r_{x_{s}}^{1D}=\overset{K}{\underset{i=1}{\sum }}W_{s_{i}}^{1D}\nu_{s_{i}}$$

(12) $$r_{y_{t}}^{CAP}=\overset{K}{\underset{i=1}{\sum }}W_{t_{i}}^{1D}\nu_{t_{i}}$$

where $W_{s_{i}}^{1D}$ and $W_{t_{i}}^{1D}$ are the weights obtained from memory querying. Like memory querying, we apply memory responding to all the threads to obtain responses from different memory representation subspaces.

Considering possible noise responses, we first concatenate single-modal features with their associated responses. A linear layer is then applied to fuse unimodal features and cross-modal vectors. This research makes it possible to focus on essential differences and filter out noisy signals. The process is defined as:


(13) $$G_{s}=FC\left(Concat\left(x_{s},r_{x_{s}}\right)\right)$$

(14) $$G_{t}=FC\left(Concat\left(y_{t},r_{y_{t}}\right)\right)$$

where FCdenotes the fully connected layer, and Concat is the concatenating function.

## 4. Experiments

### 4.1. Datasets and evaluation

We evaluate our proposed CCMN-SEN and compare it to other methods on the Hybrid Network (HybridNet; Yu et al., 2021) benchmark, a representative video-based commonsense captioning dataset (Fang et al., 2020) containing 121,618 captions for 9,721 video scenes. The dataset is divided into two parts: a training set with 6,819 videos and 85,100 captions and a test set with 2,903 videos and 36,518 captions. We follow this data partitioning in all experiments. We measure the performance of our proposed model by Meteor (Banerjee and Lavie, 2005), Rouge (Lin, 2004), CIDEr (Vedantam et al., 2015), and BLEU (*n* = 1–4; Papineni et al., 2002) for two sub-tasks according to the experimental settings in previous studies (Venugopalan et al., 2015; Gao et al., 2017; Zhou et al., 2018; Fang et al., 2020; Yu et al., 2021).

### 4.2. Implementation details

We use a single server with an NVIDIA TITAN RTX 2080Ti card for all experiments. We implement our model through the PyTorch deep learning framework and Python 3.6. We use Nvidia CUDA 11.0 and cudnn8.0 for acceleration. Our decoder is a lightweight transformer decoder consisting of 6 transformer blocks, each with eight attention heads, to ensure consistency with the experimental setup of previous work. During training, we set the batch size of one GPU to 16 and used the Adam (Kingma and Ba, 2014) optimizer with a warm-up of 5,000 steps. When initializing the memory matrix, ResNet152 (He et al., 2016) extracts 2048-dimensional appearance features, SoundNet (Aytar et al., 2016) and I3D (Carreira and Zisserman, 2017) extract 1,024-dimensional audio features and motion features, respectively, and finally, Bert (Devlin et al., 2018) extracts 768-dimensional sentimental features and extracts 768-dimensional text features. During the test, we tried different parameters and chose the best one. The number of K-Means clusters $N_{P}$ is set to 20, too many K-means clusters will make the memory vectors too similar, resulting in a performance degradation; On the contrary, if the number is set too little, it cannot guarantee enough memory vectors. The video type has 20 categories, and the number of memory vectors for each category to 20, so for the memory matrix in CCMN, the number of its memory vectors is set to 20×20=400, and the dimension is set to 512. We only select 15 most similar vectors for memory response. Selecting too many memory vectors for memory response may introduce noise, while too few may be unable to learn relevant information.

### 4.3. Results and comparisons

Table 1 shows our experimental results on the V2C dataset, which we compared to other state-of-the-art methods to demonstrate the superiority of our model. Previous research has only used memory modules on the decoder and cannot remember features across modules. However, our CCMN-SEN can align features using a shared memory matrix as a medium.

**Table 1:** Evaluation of V2C completion task using CIDER, BLEU, Rouge, and Meteor metrics.

Relation	Model	CIDER	BLEU-1	BLEU-2	BLEU-3	BLEU-4	METEOR	ROUGE-L
Attribute	S2VT (Venugopalan et al., 2015)	–	35.9	–	–	–	–	–
	Attention-Enc-Dec (Gao et al., 2017)	–	38.3	–	–	–	–	–
	Dense Captioner (Zhou et al., 2018)	–	46.0	–	–	–	–	–
	Video CMS Transformer (Fang et al., 2020)	–	47.3	–	–	–	–	–
	HybridNet (Yu et al., 2021)	–	58.7	–	–	–	–	–
	(ours)CCMN-SEN	–	**59.8**	–	–	–	–	–
Effect	S2VT (Venugopalan et al., 2015)	28.3	24.9	18.6	16.2	14.3	15.4	22.1
	Attention-Enc-Dec (Gao et al., 2017)	29.5	26.5	19.4	18.8	15.1	17.5	23.9
	Dense Captioner (Zhou et al., 2018)	36.9	33.7	24.8	21.0	20.2	20.0	29.9
	Video CMS Transformer (Fang et al., 2020)	37.3	34.8	25.9	22.5	20.4	20.8	30.6
	HybridNet (Yu et al., 2021)	66.2	49.0	42.9	40.3	38.3	30.0	41.5
	(ours)CCMN-SEN	**73.5**	**51.4**	**45.9**	**43.3**	**41.7**	**32.0**	**43.8**
Intention	S2VT (Venugopalan et al., 2015)	51.8	48.4	39.9	34.3	26.4	23.3	44.3
	Attention-Enc-Dec (Gao et al., 2017)	52.1	51.1	42.6	35.5	28.2	24.3	48.0
	Dense Captioner (Zhou et al., 2018)	60.3	59.3	47.0	37.3	31.5	28.0	53.1
	Video CMS Transformer (Fang et al., 2020)	62.0	60.8	48.4	39.1	34.1	28.5	54.6
	HybridNet (Yu et al., 2021)	92.6	69.4	60.5	55.4	53.1	35.8	60.1
	(ours)CCMN-SEN	**99.1**	**71.3**	**62.7**	**57.7**	**55.6**	**37.3**	**62.1**

We use only BLEU-1 to evaluate the attribute generation since the average length of the ground truth is just <2. The best values are highlighted in bold.

For comparison, we use HybridNet benchmark model results. Our CCMN-SEN achieves decent improvements in all evaluation metrics compared to other state-of-the-art methods, demonstrating its effectiveness and superiority. Regarding attribute performance, our CCMN-SEN outperforms HybridNet by 1.1% on BLEU-1. Our model improves in all areas, especially the CIDER metric, in the Effect and Intention sections. Our CCMN-SEN enhanced by 7.3%, 2.4%, 3%, 2.9%, 2%, and 2.3% in seven indicators (i.e., CIDER, BLEU 1-4, Meteor, and Rouge), respectively; in the Intention section, our CCMN-SEN increased by 6.5%, 1.9%, 2.2%, 2.3%, 1.5%, and 2%. Because CCMN-SEN and HybridNet both use similar structures and multi-commonsense learning, we can attribute our improved performance to multi-modal interaction and the addition of sentimental factors. We introduce sentimental features in a novel way to aid in generating commonsense captions, and we can unify cross-modality features by aligning features using a shared memory matrix. This improvement demonstrates that our CCMN-SEN is a robust baseline model for the V2C task.

**Figure 5:**
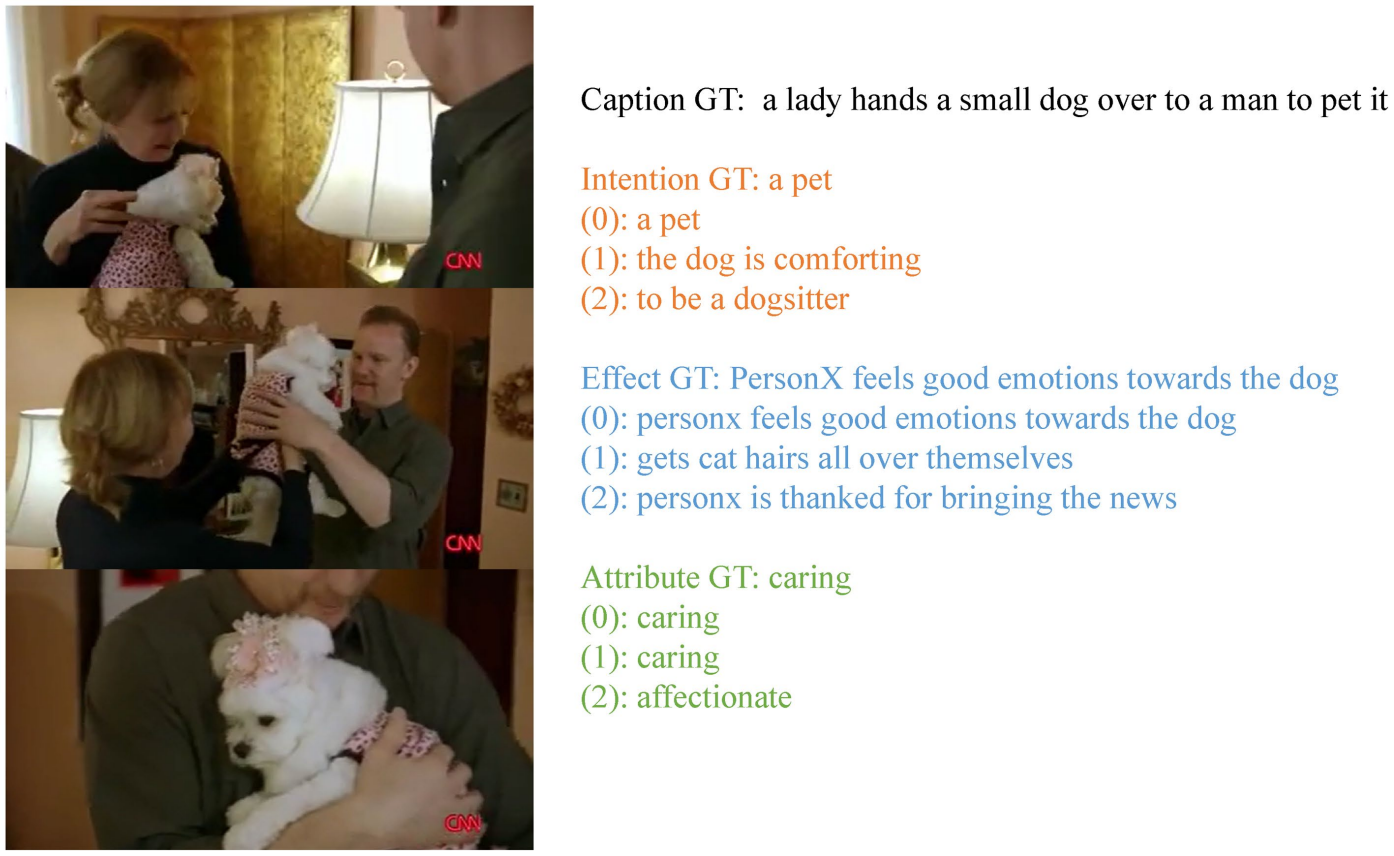
Examples of outputs for the Completion tasks along with the ground truth (GT) caption. The (0)–(2) denote the prediction results of our CCMN-SEN, HybridNet and Video2Commonsense, respectively.

An example visualization is shown in Figure 5, showing comparative results on completed tasks to illustrate the strength of our model. Our model can predict more precise intention results in the completion task, like “a pet,” compared to other methods. Other approaches deviate from the correct expression of intention (e.g., the dog is comforting). As we can see, our model can also predict more precise and effective results, such as “person X feels good emotions towards the dog.” It is not the fuzzy expression without specific sentiment predicted by other methods (e.g., gets cat hair all over themselves). The advantage can be attributed to our model’s class-dependent cross-modal module and sentimental features’ introduction, which better capture interactions between cross-modalities, capture sentimental information, and generate better reports. In contrast, other models tend to ignore sentimental details in videos.

### 4.4. Ablation studies

We conduct a detailed ablation study by examining the effectiveness of each proposed component in Table 2. We study the following models:

- **BASE:** We use the HybridNet model as the baseline for comparison, i.e., including the memory module but only considering inter-text interactions.
- **BASE + CCMN:** Based on the BASE model, we replace the standard memory module with a Class-dependent Cross-modal Memory Network module (CCMN), which considers the interaction between different modalities.
- **BASE + SEN:** Based on the BASE model, we only input sentimental features without changing the memory module and explore the impact of sentimental features on commonsense learning.
- **CCMN-SEN:** Integrates a Class-dependent and Cross-modal Memory Network considering SENtimental features (CCMN-SEN) model for sentimental control.

**Table 2:** Ablation study of CCMN-SEN model performance on the completion task.

Relation	Model	CIDER	BLEU-1	BLEU-2	BLEU-3	BLEU-4	METEOR	ROUGE-L
Attribute	BASE	–	58.7	–	–	–	–	–
	BASE + SEN	–	59.0	–	–	–	–	–
	BASE + CCMN	–	59.2	–	–	–	–	–
	CCMN-SEN	–	**59.8**	–	–	–	–	–
Effect	BASE	66.2	49.0	42.9	40.3	38.8	30.0	41.5
	BASE + SEN	69.2	49.9	44.1	41.5	39.6	31.4	42.5
	BASE + CCMN	71.5	51.1	45.3	42.7	41.0	31.8	43.3
	CCMN-SEN	**73.5**	**51.4**	**45.9**	**43.3**	**41.7**	**32.0**	**43.8**
Intention	BASE	92.6	69.4	60.5	55.4	53.1	35.8	60.1
	BASE + SEN	93.2	69.8	61.2	56.1	54.0	36.1	60.7
	BASE + CCMN	95.5	70.8	62.2	57.2	55.1	36.8	61.2
	CCMN-SEN	**99.1**	**71.3**	**62.7**	**57.7**	**55.6**	**37.3**	**62.1**

The best values are highlighted in bold.

#### 4.4.1. Effect of SENtimental features

The introduction of sentimental features increases BLEU-1 by 0.4% in terms of intention, 0.9% in effect, and 0.3% in the attribute. At the same time, we find that introducing sentimental features achieves better results on effect completion. As shown in Figure 5, our model can better recognize sentiment, demonstrating the importance of introducing sentimental features in the video-based commonsense captioning task.

#### 4.4.2. Effect of Class-dependent Cross-modal Memory Network module

We observe that CCMN can significantly improve all completion task metrics. For example, compared with the baseline model, the advantage of our CCMN can significantly increase the attention by 1.4% (69.4% vs. 70.8%), increase the effect by 2.1% (49.0% vs. 51.1%), and increase the attribute by 0.5% (58.7% vs. 59.2%) on the BLEU-1 indicator. This enhancement can be attributed to the well-learned CCMN better capturing the cross-modal information flow and embedding the information into the feature learning process. These significant improvements demonstrate the feasibility and effectiveness of our CCMN on video-based commonsense completion tasks. It is worth mentioning that the interaction between the same category of video and text will reduce noise. However, when a category always appears with other categories, other categories may scatter the matrix of related categories.

## 5. Conclusion

In this paper, we propose a Class-dependent and Cross-modal Memory Network considering SENtimental features (CCMN-SEN) framework to improve video-based commonsense caption generation by incorporating sentimental features and a class-dependent cross-modal memory network. Its sentimental features can help the model generate texts that match the video’s sentiments in less time. Then, the class-dependent cross-modal memory network applies the memory network to both the encoder and the decoder simultaneously and stores the features of different modalities through a matrix to form a video and map between texts, better-aligned features. On the V2C dataset, our CCMN-SEN achieves state-of-the-art performance, demonstrating the effectiveness and superiority of our model.

## Data availability statement

Publicly available datasets were analyzed in this study. This data can be found at: V2C dataset https://drive.google.com/file/d/1qt0JsOAqBsdCTlDUw0gw7c_IosShysoW/view.

## Author contributions

HX, YZ, JL, and YC took part in the discussion of the work described in this paper. HX wrote the first version of the paper and did part of the experiments of the paper. YZ performed the data processing and analysis. JL and YC revised the paper in different versions of the paper. All authors contributed to the article and approved the submitted version.

## Funding

This work was supported by the Humanities and Social Science Project of Ministry of Education of China (grant no. 21YJCZH186) and the National Natural Science Foundation of China (grant nos. 72171004 and 71901014).

## Conflict of interest

The authors declare that the research was conducted in the absence of any commercial or financial relationships that could be construed as a potential conflict of interest.

## Publisher’s note

All claims expressed in this article are solely those of the authors and do not necessarily represent those of their affiliated organizations, or those of the publisher, the editors and the reviewers. Any product that may be evaluated in this article, or claim that may be made by its manufacturer, is not guaranteed or endorsed by the publisher.
